# Behavior control in the sensorimotor loop with short-term synaptic dynamics induced by self-regulating neurons

**DOI:** 10.3389/fnbot.2014.00019

**Published:** 2014-05-23

**Authors:** Hazem Toutounji, Frank Pasemann

**Affiliations:** Department of Neurocybernetics, Institute of Cognitive Science, University of OsnabrückOsnabrück, Germany

**Keywords:** sensorimotor loop, autonomous agent, synaptic plasticity, short-term plasticity, homeostasis, self-regulation, hysteresis, oscillation

## Abstract

The behavior and skills of living systems depend on the distributed control provided by specialized and highly recurrent neural networks. Learning and memory in these systems is mediated by a set of adaptation mechanisms, known collectively as neuronal plasticity. Translating principles of recurrent neural control and plasticity to artificial agents has seen major strides, but is usually hampered by the complex interactions between the agent's body and its environment. One of the important standing issues is for the agent to support multiple stable states of behavior, so that its behavioral repertoire matches the requirements imposed by these interactions. The agent also must have the capacity to switch between these states in time scales that are comparable to those by which sensory stimulation varies. Achieving this requires a mechanism of short-term memory that allows the neurocontroller to keep track of the recent history of its input, which finds its biological counterpart in short-term synaptic plasticity. This issue is approached here by deriving synaptic dynamics in recurrent neural networks. Neurons are introduced as self-regulating units with a rich repertoire of dynamics. They exhibit homeostatic properties for certain parameter domains, which result in a set of stable states and the required short-term memory. They can also operate as oscillators, which allow them to surpass the level of activity imposed by their homeostatic operation conditions. Neural systems endowed with the derived synaptic dynamics can be utilized for the neural behavior control of autonomous mobile agents. The resulting behavior depends also on the underlying network structure, which is either engineered or developed by evolutionary techniques. The effectiveness of these self-regulating units is demonstrated by controlling locomotion of a hexapod with 18 degrees of freedom, and obstacle-avoidance of a wheel-driven robot.

## 1. Introduction

Living systems, which have to survive in a complex, permanently changing environment must exhibit a life-sustaining behavior. For autonomous agents, such as *animats*, this is one of the desired capacities. For achieving this objective, autonomous agents are equipped with different types of sensors, with proprioceptors monitoring their internal states, and with motors to articulate their body movements. In addition, since every movement of the body changes the inputs to the sensors and proprioceptors, these agents always operate in a sensorimotor loop.

Even when the overall task is apparently simple, autonomous agents are still expected to express diverse behavior in order to accomplish the task, and the rich dynamics provided by artificial recurrent neural networks is usually invoked for the control of this behavior. Examples include tropisms of wheel-driven robots (Hülse and Pasemann, 2002; Smith et al., 2002), biped walking (Manoonpong et al., 2007; Kubisch et al., 2011), active tracking (Negrello and Pasemann, 2008), quadruped locomotion, (Manoonpong et al., 2006; Ijspeert et al., 2007; Shim and Husbands, 2012), hexapod locomotion (Beer and Gallagher, 1992), and swimming robots (Ijspeert et al., 2007; Shim and Husbands, 2012).

The ability of recurrent neurocontrollers to generate successful behavior depends highly on its connectivity structure as well as on the synaptic efficacies of its connections. Suitable neurocontrollers are usually found by evolutionary techniques (Nolfi and Floreano, 2000). However, synaptic plasticity and regulatory mechanisms of neural activity constitute the biological basis for learning and memory (Cooper et al., 2004), and were taken up by (evolutionary) robotics as a tool for adding learning abilities to autonomous agents (Nolfi and Floreano, 1999; Di Paolo, 2000; Smith et al., 2002; Williams and Noble, 2007; Vargas et al., 2009; Santos et al., 2010; Hoinville et al., 2011). Incorporating plasticity in the neural control of robots takes the load off evolution for finding the right synaptic weights and/or operating range of the neurons within the network, and limits the role of the evolutionary process to the allocation of suitable connectivity structure, which considerably reduces the search space.

We follow on the lead of these studies, where we assume that the connectivity structure is given as a result of an evolutionary process, and we concentrate on deriving synaptic dynamics for the neural control of artificial agents acting in the sensorimotor loop. Our model is referred to as the *self-regulating neuron*, or the SR-neuron, for short. A similar model was first proposed for a slightly different synaptic dynamics and another neuron type (Zahedi and Pasemann, 2007). The SR-neuron differs from the previous approaches in that its synaptic dynamics acts on a faster time scale. Here, synaptic efficacies do not change due to a slow adaptation process based on repetition of pre- and postsynaptic activity patterns. Instead, they adapt to sensory stimulations at the rate by which these stimulations change. This feature makes the SR-neurons suitable for the requirement of real-time diverse dynamic behavior and for a quick reaction to varying stimuli. As such, and unlike previous studies, the SR-neuron dynamics does not aim at augmenting the neurocontroller with learning, in the sense of a gradual change of behavior to a better one. In other words, there is no training phase that ends with higher fitness and a steady state of the synaptic weights. Instead, synaptic weights are constantly adapting in response to the changes of external stimuli.

The synaptic dynamics of the self-regulating neuron does not replicate a particular plasticity mechanism that is empirically observed in biological systems. Nevertheless, it is *biologically-inspired* in three different ways, by which it exploits the functional properties of biological plasticity for the benefit of a stable and successful behavior of an artificial agent.

First, self-regulating neurons act as *homeostatic* elements, which try to maintain one of two desired activity states, one referring to low, and the other to high activity. Homeostatic regulation is only necessary to operate when the system is confronted with some external perturbations. Since recurrent neurocontrollers of artificial agents have to work in the sensorimotor loop, they are permanently driven by continuously changing sensory inputs. A neural mechanism for homeostatic plasticity should therefore lead to a stabilization of behavior, by providing the controller with the means necessary to cope with these fast varying sensory inputs.

Biological findings strongly support the existence of such mechanisms, where the incoming signals to a neuron (Davis and Goodman, 1998), or the neuron's own excitability (Turrigiano and Nelson, 2004) is homeostatically adjusted to match a functionally desirable neural activation, such as maximizing the entropy of the neural output (Triesch, 2007; Marković and Gros, 2012). Many models from theoretical neuroscience incorporate homeostatic plasticity mechanisms in recurrent neural networks, either in the form of *synaptic scaling* of afferents (Remme and Wadman, 2012; Zenke et al., 2013), *intrinsic plasticity* of neural excitability (Lazar et al., 2007; Marković and Gros, 2012; Naudé et al., 2013; Toutounji and Pipa, 2014), or both (Lazar et al., 2009; Zheng et al., 2013). These mechanisms also find their application in improving time series prediction in echo state networks (Schrauwen et al., 2008). Homeostasis has also been discussed in the context of adaptation and learning in cybernetics (Ashby, 1960), and there are many examples of its successful contribution to learning in recurrent neural control of robots (Di Paolo, 2000; Hoinville and Hénaff, 2004; Williams and Noble, 2007; Vargas et al., 2009; Santos et al., 2010; Hoinville et al., 2011).

Second, the synaptic dynamics of the self-regulating neuron partially adheres to Hebb's postulate (Hebb, 1949), where the synapses between mutually active neurons are potentiated. Homeostasis, however, prevents the overgrowth of synaptic weights due to the constant potentiation in a fashion similar to the *BCM theory* (Bienenstock et al., 1982), and its spiking neurons variants (Toyoizumi et al., 2005; Clopath et al., 2010). In robotics, learning with a variant of Hebbian plasticity is demonstrated, for example, by Harter and Kozma (2005); Santos et al. (2010); Hoinville et al. (2011).

While these studies favor steady-state synaptic weights, controlled bifurcations of neural dynamics might be very desirable in the context of the sensorimotor loop (Ashby, 1960). During the autonomous agent's lifespan, it is important that changes in its stimulation elicit history-dependent responses, which entails a form of *working memory* for the agent (Negrello and Pasemann, 2008). The importance of this functionality comes from the fact that environmental cues are themselves temporally extended (Buonomano and Maass, 2009; Toutounji and Pipa, 2014). As such, an autonomous agent's behavior must come as a response to these temporally extended stimuli, rather than to instantaneous states of its environment. This directly connects to the third point of relatedness to biological plasticity, that is, *short-term plasticity* (Zucker and Regehr, 2002; Abbott and Regehr, 2004). Due to short-term plasticity, synaptic efficacy changes on faster time scales in ways that reflect the history of the presynaptic activity. This history-dependence may mediate working memory in recurrent neural networks (Mongillo et al., 2008). The self-regulating neuron exhibits this history-dependence, where changes in temporally extended stimuli are captured by the fast synaptic dynamics. This synaptic dynamics then controls the neuron's bifurcation between the two desired activity states, which leads to history-dependent adjustment of behavior.

Here, it is shown that self-regulating neurons are suitable for the control of an autonomous agent's behavior under the sensory perturbations of the sensorimotor loop. The activity of neurons, together with the synaptic efficacies, change over time, but usually fluctuate around some average values, as has been demonstrated for simple examples in Pasemann (2013). A self-regulating neuron is able to attain and maintain a desirable level of activity even if it is confronted with unpredictable, and more or less severe perturbations, induced by changing sensory inputs. Furthermore, it has different internal states at its disposal, leading to different stable behaviors, which may be appropriate for one or the other external situation.

The following section introduces self-regulating neurons, together with the properties of the induced synaptic plasticity rule. Because these self-regulating neurons have to operate as elements of neurocontrollers in the sensorimotor loop, the synaptic weights of these neurocontrollers change dynamically according to sensory stimuli or internal feedback loops. With this in mind, the dynamics of simple neural modules is analyzed next under varying stimulation, so as to reach a basic understanding of the stability properties of these modules. This is followed by discussing examples of successful control of behavior for synchronizing coupled reflex loops, for locomotion of a hexapod walking machine, and for obstacle-avoidance of a wheel-driven robot.

## 2. Self-regulating neurons

Given a neural network *N* with *n* neurons, and a structure matrix *c*, defined by *c*_*ij*_ = +1 (−1) for an excitatory (inhibitory) connection from neuron *j* to neuron *i* and *c*_*ij*_ = 0, otherwise. A single self-regulating neuron *i* is described as a parameterized discrete-time 3-dimensional dynamical system with state variables (*a*_*i*_, ξ_*i*_, η_*i*_) ∈ ℝ × ℝ^+^ × ℝ^+^ for *i* = 1, …, *n*, where *a*_*i*_ denotes its activation, and ξ_*i*_ and η_*i*_ its *receptor* and *transmitter* strength, respectively. Furthermore, it may have a bias value θ_*i*_ that is the sum of a constant bias θ_*i*_ and an external drive *I*. The output *o*_*i*_ = τ(*a*_*i*_) of a neuron *i* is given by the sigmoidal hyperbolic tangent transfer function τ := tanh. The weight *w*_*ij*_ of the connection from neuron *j* to neuron *i* is then defined by

(1) $$w_{ij}:=c_{ij}\xi_{i}\eta_{j}.$$

We assume that there exists a desirable state a^*^_i_ for the activation of a neuron, and that the 3-dimensional dynamics is to be defined so as to stabilize this state for a certain range of input signals. Such a state defines a preferred operational range of the neurons' dynamics. There are two canonical choices for such a desirable state. One is for the neuron to operate around the *linear* domain of the transfer function, i.e., a^*^_i_ = 0 for the hyperbolic tangent nonlinearity. However, recurrent neural networks are expected to capture and respond to environmental stimuli that are riddled by *nonlinear* dependencies. As such, it is reasonable to enforce the nonlinear properties of recurrent neural networks, in order for them to reflect, in their activity, these nonlinear environmental conditions. Therefore, the desired state in the following corresponds to an activation a^*^_i_ for which the nonlinearity of the transfer function τ is “maximal,” i.e., its third derivative satisfies τ‴(a^*^) = 0. Since τ is an antisymmetric function, its third derivative τ‴ is symmetric, and there are two such *operating points* satisfying this condition and they take values

$$a^{*}:=a_{\pm }^{*}\approx \pm 0.658479\text{ and }\tau (a^{*})=\pm \sqrt{\frac{1}{3}}\approx \pm 0.5773503.$$

This means that a neuron prefers a high or low state of activity, or, in terms of rate models, a high or low firing rate.

The basic equations for the dynamics are then set up as follows. The standard additive discrete-time dynamics for the activation *a*_*i*_ of a neuron is given by

(2) $$\begin{array}{l} a_{i}(t+1)={\bar{\theta }}_{i}+\xi_{i}(t)\;\sum_{j=1}^{n}c_{ij}\;\eta_{j}(t)\tau (a_{j}(t))\\ \text{ where }i=1,\ldots ,n. \end{array}$$

Furthermore, it is assumed that the receptor strength ξ_*i*_ and the transmitter strength η_*i*_ for *i* = 1, …, *n* are both *positive* for all times. The dynamics of the receptor strength ξ_*i*_ modulates the incoming signals to the neuron such that its response becomes maximally nonlinear. In other words, the receptor strength is responsible for pushing the activation *a*_*i*_ of the neuron toward one of the operating points a^*^_±_, and is given by

(3) $$\begin{array}{l} \xi_{i}(t+1)=\xi_{i}(t)[1+\beta \cdot (\tau^{2}(a^{*})-\tau^{2}(a_{i}))]\\ \text{ where }0<\beta <1. \end{array}$$

The transmitter strength η_*i*_ communicates the neuron's activity to its targets, i.e., it increases with the activation *a*_*i*_ of the neuron. It also has a decay rate (1 − γ), which is necessary for the convergence of the dynamics, as we show later. Thus, the transmitter dynamics is defined by

(4) $$\eta_{i}(t+1)=(1-\gamma )\;\eta_{i}(t)+\delta [1+\tau (a_{i})]\text{ where }0<\gamma ,\delta <1.$$

The discrete-time dynamics *f* : ℝ × ℝ^+^ × ℝ^+^ → ℝ × ℝ^+^ × ℝ^+^ given by Equations (2-4) is called the dynamics of self-regulating neurons or *SRN-dynamics*, for short.

The weight change per time step is then given by

(5) $$\begin{array}{l} \Delta w_{ij}(t)=w_{ij}(t+1)-w_{ij}(t)\\ \;=c_{ij}(\xi_{i}(t+1)\eta_{j}(t+1)-\xi_{i}(t)\eta_{j}(t)). \end{array}$$

Replacing ξ_*i*_(*t* + 1) and η_*j*_(*t* + 1) by their dynamics from Equations (3,4) leads to

(6) $$\Delta w_{ij}(t)=c_{ij}w_{ij}(t)\cdot [F(a_{i}(t))+G(a_{j}(t))+H(a_{i}(t),a_{j}(t))],$$

where

(7) $$\begin{array}{l} \;F(a_{i})=-\gamma +\beta (1-\gamma )(\tau^{2}(a^{*})-\tau^{2}(a_{i})),\\ \;G(a_{j})=\frac{\delta }{\eta_{j}}(1+\tau (a_{j})),\\ H(a_{i},a_{j})=\frac{\beta \delta }{\eta_{j}}(1+\tau (a_{j}))(\tau^{2}(a^{*})-\tau^{2}(a_{i})). \end{array}$$

This demonstrates two of the biologically-inspired features of the synaptic dynamics. The weight change depends on the product of the presynaptic and postsynaptic activations through the (anti-)Hebbian element *H*(*a*_*i*_, *a*_*j*_), which includes the term τ(*a*_*j*_)τ^2^(*a*_*i*_). In addition, the term *H*(*a*_*i*_, *a*_*j*_) is not always positive, since its sign depends on the postsynaptic activity *a*_*i*_. When |*a*_*i*_| < |a^*^|, the term is positive which leads to Hebbian-like synaptic potentiation. Otherwise, the term is negative and the synaptic efficacy is depressed in an anti-Hebbian fashion. In other words, the term (τ^2^(a^*^) − τ^2^(*a*_*i*_)) reflects the postsynaptic-dependent homeostatic nature of the synaptic dynamics, where a regime of potentiation is separated from a regime of depression at the threshold a^*^.

## 3. Results

In what follows, we rigorously analyze the dynamics of simple self-regulating neural modules. Namely, we study the stable dynamics of a SR-neuron without self connection. We then prove that a SR-neuron with an excitatory self-connection is bistable under certain conditions, which confirms observations that were made in Pasemann (2013). We show in addition that a SR-neuron with inhibitory self-connection oscillates with period-2. We finally demonstrate the operation of networks of these modules for the control of behavior in the sensorimotor loop.

### 3.1. Dynamics of self-regulating neurons

To get a first impression of the SRN-dynamics we study the dynamics of a single neuron with and without self-connection. Suppressing the neuron's index *i*, the 3-dimensional dynamics reads

(8) $$\begin{array}{l} a(t+1)=\theta +c\xi (t)\eta (t)\tau (a(t))+\xi I(t),\\ \xi (t+1)=\xi (t)[1+\beta \cdot (\tau^{2}(a^{*})-\tau^{2}(a))],\\ \eta (t+1)=(1-\gamma )\;\eta (t)+\delta [1+\tau (a)], \end{array}$$

where *I* represents the inputs coming from other neurons, i.e.,

(9) $$I(t):=\sum_{j\neq i}c_{j}\eta_{j}\tau (a_{j}(t)).$$

For the moment, we assume that *I* is constant over time, and that there exists a stable fixed point (a^*^, ξ^*^, η^*^) of the 3-dimensional SRN-dynamics, in order to derive conditions for its existence. Throughout this section, the parameters β, γ, and δ are set to 0.1. To determine the stability of the dynamical system (Equation 8) at a fixed point (a^*^, ξ^*^, η^*^), we study its linearization at a state (*a*, ξ, η) ∈ ℝ × ℝ^+^ × ℝ^+^, which is given by the Jacobian matrix

(10) $$(Df)(a,\xi ,\eta )=\left(\begin{array}{c} \begin{array}{ccc} c\xi \eta (1-\tau^{2}) & c\eta \tau +I & c\xi \tau \\ -2\beta \xi \tau (1-\tau^{2}) & 1+\beta (\tau^{2}(a^{*})-\tau^{2}(a)) & 0\\ \delta (1-\tau^{2}) & 0 & 1-\gamma \end{array} \end{array}\right).$$

There are three possible fixed points for the dynamical system (Equation 8). These are the two *desirable* fixed points *x*_±_ = (a^*^_±_, ξ^*^_±_, η^*^_±_) with transmitter strength $\eta_{\pm }^{*}=\frac{\delta }{\gamma }(1+\tau (a_{\pm }^{*}))$, and the *trivial* fixed point *x*_0_ = (θ, 0, η_0_) with a vanishing receptor strength, and a transmitter strength $\eta_{0}=\frac{\delta }{\gamma }(1+\tau (\theta ))$. We refer to the last situation as a “dead neuron,” because it is not able to process incoming signals. Whether one of these fixed points is asymptotically stable or not depends on the eigenvalues of (*Df*) (a^*^, ξ^*^, η^*^), as we show next.

#### 3.1.1. Dynamics without self-connection

For a first analysis, we study a single neuron without self-connection, i.e., *c* = 0, and with a fixed bias value θ. It is driven by the input signal *I*. The linearization of SRN-dynamics then reads

(11) $$(Df)(a,\xi ,\eta )=\left(\begin{array}{ccc} 0 & I & 0\\ -2\beta \xi \tau (1-\tau^{2}) & 1+\beta (\tau^{2}(a^{*})-\tau^{2}(a)) & 0\\ \delta (1-\tau^{2}) & 0 & 1-\gamma \end{array}\right).$$

A fixed point x^*^ is asymptotically stable if all the eigenvalues λ_*k*_ of (*Df*)(x^*^) satisfy |λ_*k*_| < 1. The two desirable fixed points *x*_±_ = (a^*^_±_, ξ^*^_±_, η^*^_±_) for this neuron also satisfy the equation

(12) $$a_{\pm }^{*}-\theta =\xi_{\pm }^{*}I.$$

**Figure 1:**
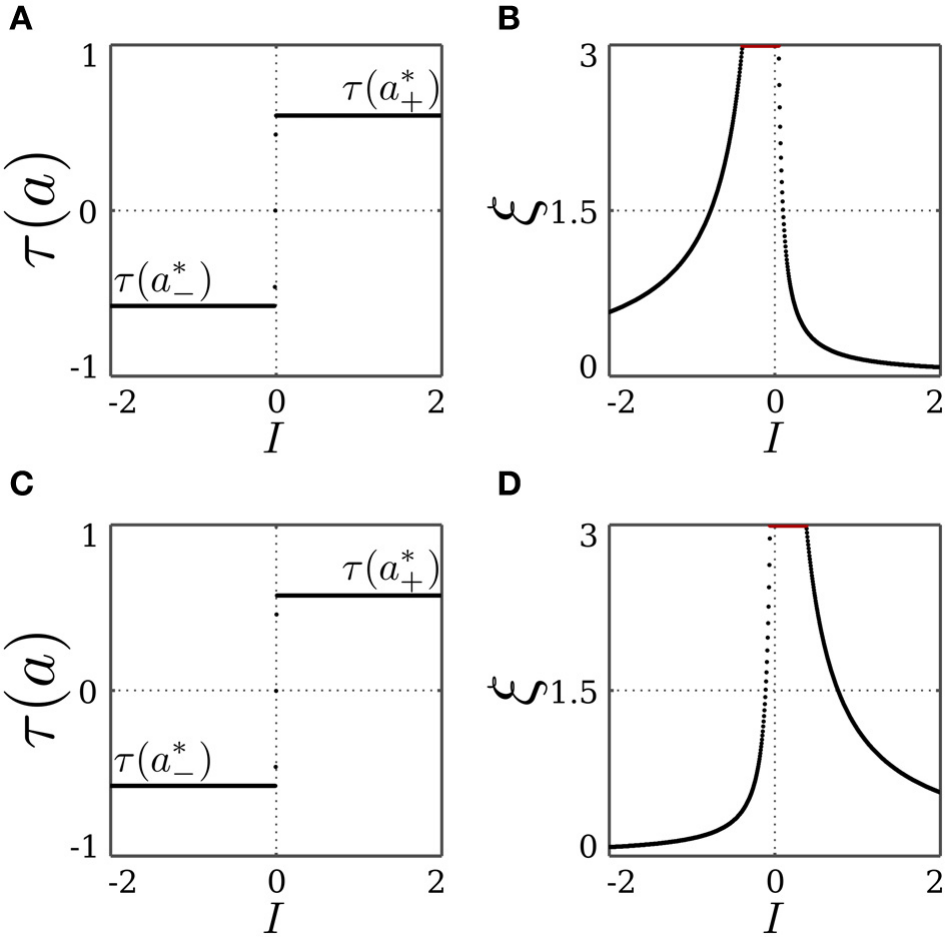
**Stable dynamics of a SR-neuron without self-connection for a homeostatic bias and varying input. (A,B)** Bifurcation diagrams of the output τ(*a*) and the receptor strength ξ for varying input *I* and a *positive* homeostatic bias θ = +0.5. **(C,D)** Bifurcation diagrams of the output τ(*a*) and the receptor strength ξ for varying input *I* and a *negative* homeostatic bias θ = −0.5.

First, one observes from condition (12) that the receptor strength ξ^*^_±_ diverges for inputs *I* → 0, and thus, *x*_±_ are both unstable when *I* = 0. Otherwise, replacing the input *I* > 0 in the linearization (Equation 11) with its value from condition (12), leads to the following eigenvalues around the fixed points *x*_+_:

(13) $$\begin{array}{l} \lambda_{1,2}(a_{+}^{*})=\lambda_{\pm }(a_{+}^{*})=\frac{1\pm \sqrt{1-8\beta (1-\tau^{2}(a_{+}^{*}))(a_{+}^{*}-\theta )\tau (a_{+}^{*})}}{2},\\ \;\lambda_{3}=1-\gamma , \end{array}$$

and similarly for *I* < 0 and the fixed point *x*_−_, but with λ_±_ being a function of a^*^_−_ instead. For both fixed points, the stability condition |λ_*k*_| < 1 always holds for λ_−_ and λ_3_. This also stresses the necessity of introducing the decay term parameterized by γ of the transmitter dynamics η for the stability of the SR-neuron, without which λ_3_ = 1. On the other hand, the stability condition only holds for λ_+_ when (a^*^_±_− θ) τ(a^*^_±_) < 0. It follows that for θ ∈ (a^*^_−_, a^*^_+_), the SR-neuron is homeostatic, i.e., one of the fixed points *x*_±_ is stable, for all inputs *I* ∈ ℝ \ {0}. We thus call a bias θ that is within the range (a^*^_−_, a^*^_+_) a *homeostatic bias*. Asymptotically, it acts like a binary neuron switching from low activity a^*^_−_ to high activity a^*^_+_ around *I* = 0. This is also confirmed by Figure 1, showing bifurcation diagrams for the output τ(*a*) and the receptor strength ξ under these conditions.

In addition, keeping in mind that a^*^_−_ = −a^*^_+_, the fixed point *x*_−_ satisfies condition (12) when *I* < 0, if θ > a^*^_+_, which leads to (a^*^_−_ − θ) τ(a^*^_−_) < 0, and as such, |λ_+_(a^*^_−_)| < 1 holds. This entails that *x*_−_ is asymptotically stable when *I* < 0 and θ > a^*^_+_. Correspondingly, *x*_+_ is asymptotically stable when *I* > 0 and θ < a^*^_−_. In other words, the SR-neuron without self-connection is homeostatic only over half of the input domain, when θ ∉ [a^*^_−_, a^*^_+_]. On the other hand, the trivial fixed point *x*_0_, corresponding to a dead neuron, becomes stable for all *I*, since the eigenvalues of (*Df*)(*x*_0_) are

(14) $$\lambda_{1}=0,\lambda_{2}=1+\beta (\tau^{2}(a_{\pm }^{*})-\tau^{2}(\theta )),\lambda_{3}=1-\gamma ,$$

which satisfy |λ_*k*_| < 1 when θ ∉ [a^*^_−_, a^*^_+_].

**Figure 2:**
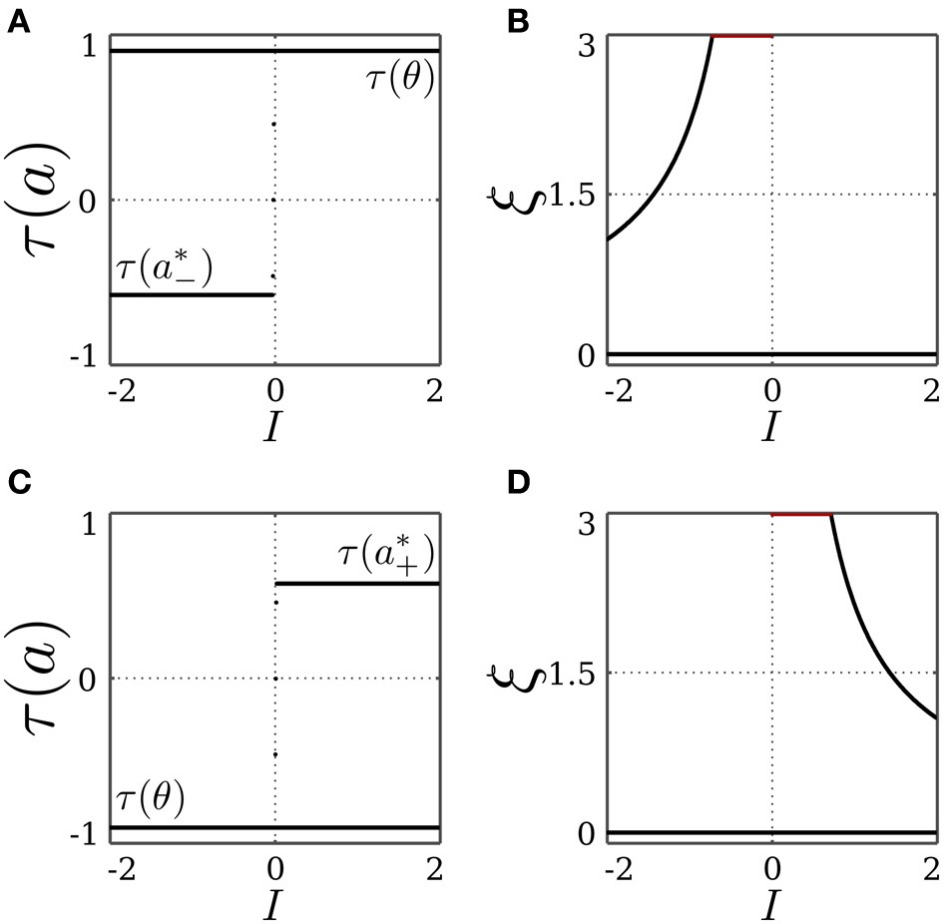
**Stable dynamics of a SR-neuron without self-connection for a non-homeostatic bias and varying input. (A,B)** Bifurcation diagrams of the output τ(*a*) and the receptor strength ξ for varying input *I* and a *positive* non-homeostatic bias θ = +1.5. **(C,D)** Bifurcation diagrams of the output τ(*a*) and the receptor strength ξ for varying input *I* and a *negative* non-homeostatic bias θ = −1.5.

To summarize, the SR-neuron without self connection and a bias θ ∉ [a^*^_−_, a^*^_+_] is bistable over half of the input domain, where one stable fixed point corresponds to the homeostatic state, and the other to the trivial state. The SR-neuron would then converge to one of the two fixed points depending on the initial conditions. On the other half of the input domain, the neuron is globally stable at the trivial fixed point. These observations are confirmed by Figure 2, showing bifurcation diagrams for the output τ(*a*) and the receptor strength ξ under these conditions.

#### 3.1.2. Trivial dynamics with self-connection

Adding a self-connection *w*:= *c* ξ η to the SR-neuron provides an additional input, so that the new input signal becomes I(t)+cητ(a(t)), where *I* again corresponds to the input from other neurons as in Equation (9).

The linearization (Equation 10) around the trivial fixed point *x*_0_ = (θ, 0, η_0_) leads to the same eigenvalues (Equation 14), regardless of whether the self connection is excitatory or inhibitory. This entails that the SR-neuron with self-connection is stable at the trivial fixed point for all *I*, when its bias is non-homeostatic, i.e., θ ∉ [a^*^_−_, a^*^_+_].

On the other hand, the linearization (Equation 10) around the desirable fixed points *x*_±_ = (a^*^_±_, ξ^*^_±_, η^*^_±_) leads to complex closed-form formulas for the eigenvalues that are of no help regarding the stability of these fixed points. However, we may rely on the 1-dimensional standard hyperbolic tangent neuron with self-connection:

(15) a(t+1)=θ+wτ(a(t)).

This neuron is parameterized by its bias θ and self-weight *w*, and, for each parameterization, its asymptotic dynamics is easy to derive. Since both neuron models, the SR-neuron and the standard neuron, share the same transfer function tanh, it is possible to infer the stability of the former from the more familiar properties of the latter, given certain bias and self-weight values, as we show next.

#### 3.1.3. Dynamics with excitatory self-connection

Suppose that the fixed points *x*_±_ = (a^*^_±_, ξ^*^_±_, η^*^_±_) for the SR-neuron with self-connection are asymptotically stable. These fixed points then satisfy

(16) $$a^{*}=\theta +\xi^{*}(I+c\eta^{*}\tau (a^{*})).$$

We start by setting θ = *I* = 0. Then, the following holds

(17) $$c\xi_{\pm }^{*}\eta_{\pm }^{*}=\frac{a_{\pm }^{*}}{\tau (a_{\pm }^{*})}\approx 1.14>0,$$

which is only true for the case of an *excitatory self-connection*, i.e., *c* = +1. For an increasing excitatory self-connection and a zero bias, the standard additive hyperbolic tangent neuron (Equation 15) undergoes a *cusp catastrophe* (Guckenheimer and Kuznetsov, 2007) at the critical point (θ_*c*_ = 0, *w_c_* = 1), and the neuron corresponds to a *bistable* system (Pasemann, 1993; Hülse and Pasemann, 2002). Because the asymptotic self-weight w^*^_±_ = ξ^*^_±_ η^*^_±_ of the SR-neuron (Equation 17) is larger than the critical value *w_c_* = 1, the SR-neuron becomes bistable as well, which allows for *hysteresis* phenomena.

The critical point (θ_*c*_ = 0, *w_c_* = + 1) belongs to the *bifurcation set* 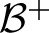, at which the standard hyperbolic tangent neuron (Equation 15) changes from being monostable to being bistable. The bifurcation set is parameterized by the bias and self-weight, and is derived in Pasemann (1993) for a standard neuron with logistic nonlinearity σ(*a*) = (1 + *e*^−*a*^)^−1^. For a hyperbolic tangent nonlinearity, 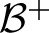 is given by

(18) $$\theta^{2}=\frac{4{\left(w-1\right)}^{3}}{9w},$$

while, at the fixed point *x*_±_, the positive self-coupling w^*^_±_ of the SR-neuron changes *linearly* with the bias according to

(19) $$w_{\pm }^{*}=\frac{a_{\pm }^{*}-\theta }{\tau (a_{\pm }^{*})}.$$

**Figure 3:**
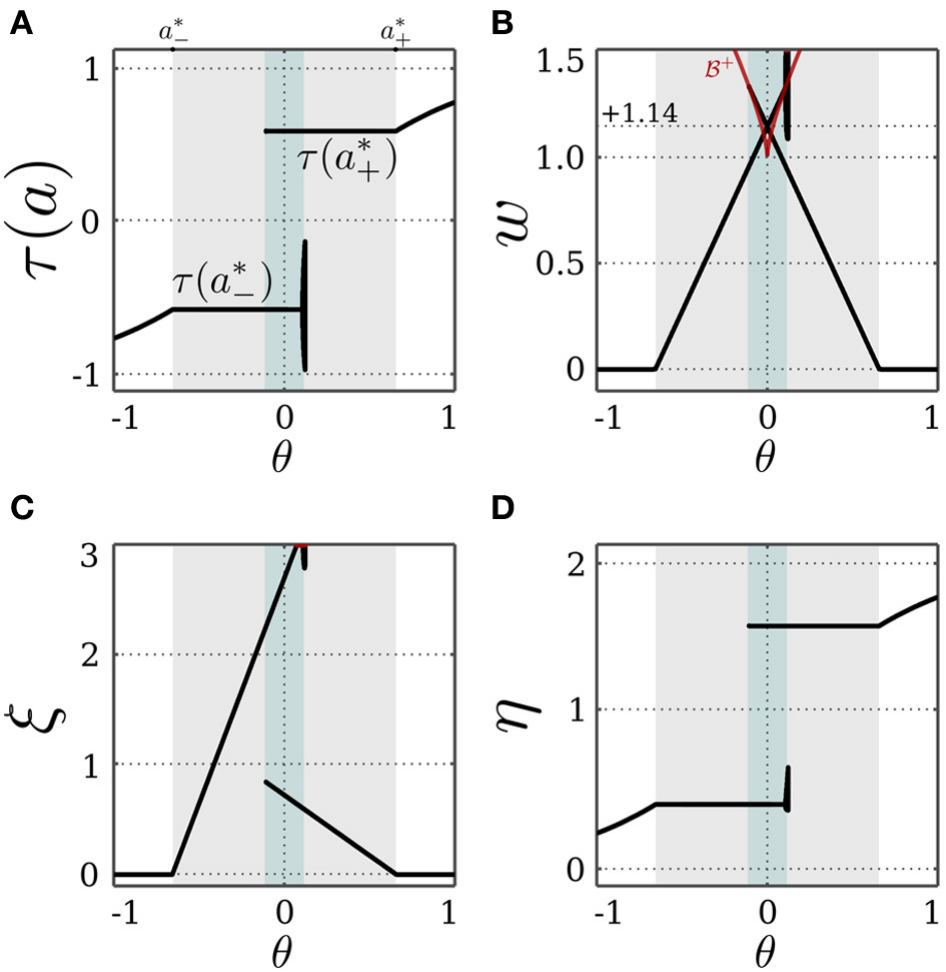
**Stable dynamics of a SR-neuron with excitatory self-connection for varying bias**. Bifurcation diagrams of **(A)** the output τ(*a*), **(B)** the *positive* self-weight *w* = +ξ η, **(C)** the receptor strength ξ, and **(D)** the transmitter strength η for varying bias θ. The gray-shaded area corresponds to the bias domain θ ∈ (a^*^_−_, a^*^_+_) at which the SR-neuron is *homeostatic*. The cyan-shaded area marks the hysteresis domain θ ∈ [−0.11, 0.11] at which the SR-neuron is *bistable*. The neuron shows a narrow range of quasi-periodic behavior when passing from a^*^_−_ to a^*^_+_. **(B)** The red curve denotes the bifurcation set 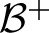 that marks the parameters domain, where a standard additive hyperbolic tangent neuron is bistable. The SR-neuron ceases from exhibiting bistability, when the positive self-coupling weight becomes lower than the bifurcation set.

The SR-neuron is bistable when w^*^_+_ or w^*^_−_ or both are above the bifurcation set 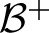. As such, the intersection of the bifurcation set 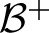 defined by Equation (18) and the self-coupling of an SR-neuron as a function of the bias in Equation (19), leads to the bias range θ ∈ [−0.11, +0.11], within which the SR-neuron is bistable. Outside of this range, both w^*^_+_ and w^*^_−_ are bellow 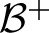, resulting in the SR-neuron becoming monostable. These findings can be verified by keeping *I* = 0 and varying the bias term θ as shown in Figure 3.

**Figure 4:**
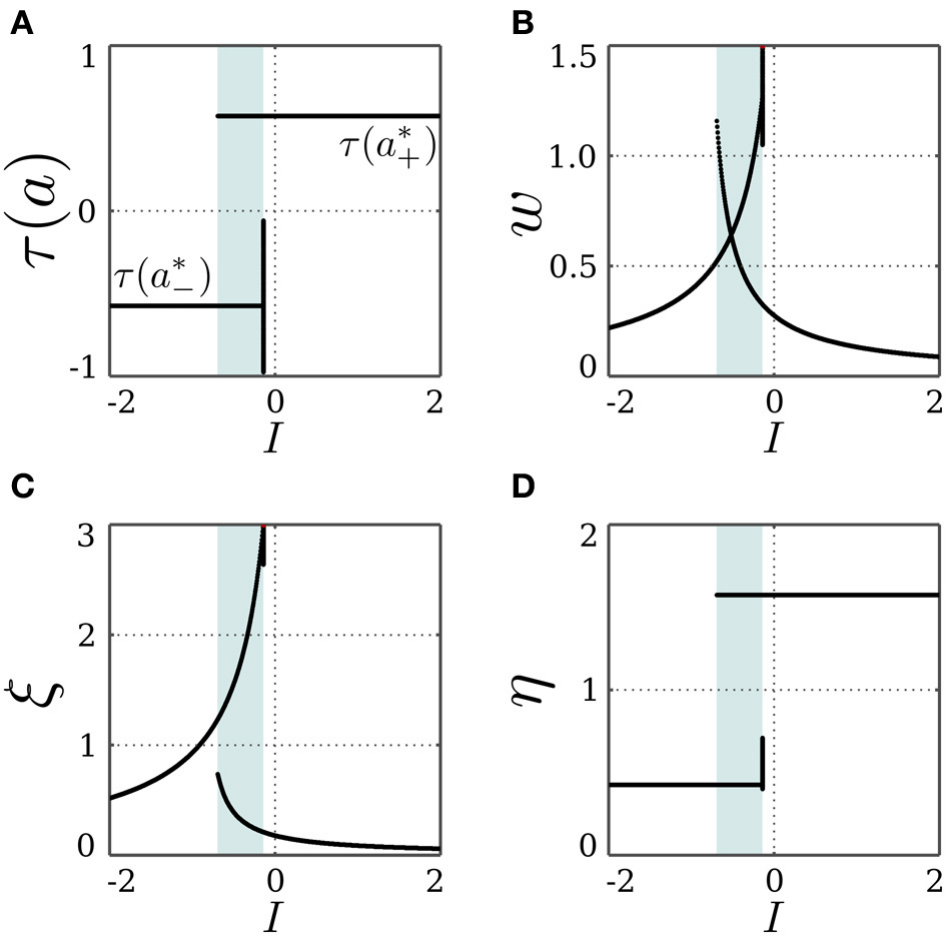
**Stable dynamics of a SR-neuron with excitatory self-connection for a homeostatic bias and varying input**. Bifurcation diagrams of **(A)** the output τ(*a*), **(B)** the *positive* self-weight *w* = +ξ η, **(C)** the receptor strength ξ, and **(D)** the transmitter strength η for varying input *I* and a *positive* homeostatic bias θ = +0.5. The cyan-shaded area marks the hysteresis domain at which the SR-neuron is *bistable*. The neuron shows a narrow range of quasi-periodic behavior when passing from a^*^_−_ to a^*^_+_.

We now assume that there exists a stationary input *I*, and that the bias θ ∈ (a^*^_−_, a^*^_+_). Under these conditions, the SR-neuron is homeostatic over the whole input domain, and it exhibits hysteresis phenomena over some input range, as is shown for θ = +0.5 in Figure 4. For a narrow input range, one observes that the SR-neuron may show quasi-periodic oscillations when passing from one operating point to the other. These oscillations depend on the bias value and the parameters β, γ, and δ.

#### 3.1.4. Dynamics with inhibitory self-connection

For an *inhibitory self-connection*, i.e., *c* = −1, and no input, Equation (16) can be solved when θ ∉ [a^*^_−_, a^*^_+_]. However, the trivial fixed point *x*_0_ is stable at this bias domain, as shown in section 3.1.2, and an inhibitory self-connection can never satisfy the bistability condition bounded from below by the bifurcation set 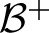 (Equation 18). This rules out the possibility for a^*^_±_ being stable, which entails that the SR-neuron is never homeostatic under these conditions. However, with no bias and a self-weight *w* ≈ −1.14, the state a^*^_+_ is mapped to a^*^_−_ and vice versa, as suggested by Equation (17). Thus, we expect a period-2 oscillation between the two states. Regarding the stability of this oscillation, we return to the standard additive hyperbolic tangent neuron (Equation 15). For an increasing inhibitory self-connection, neuron (Equation 15) undergoes a *supercritical period doubling bifurcation* at the critical point (θ_*c*_ = 0, *w_c_* = −1), and the neuron corresponds to a *period-2 oscillator*. This supports the existence of a stable period-2 oscillation for the SR-neuron when (θ = 0, *w* ≈ −1.14), since this point lies within the period-2 parameter range of a standard hyperbolic tangent neuron.

**Figure 5:**
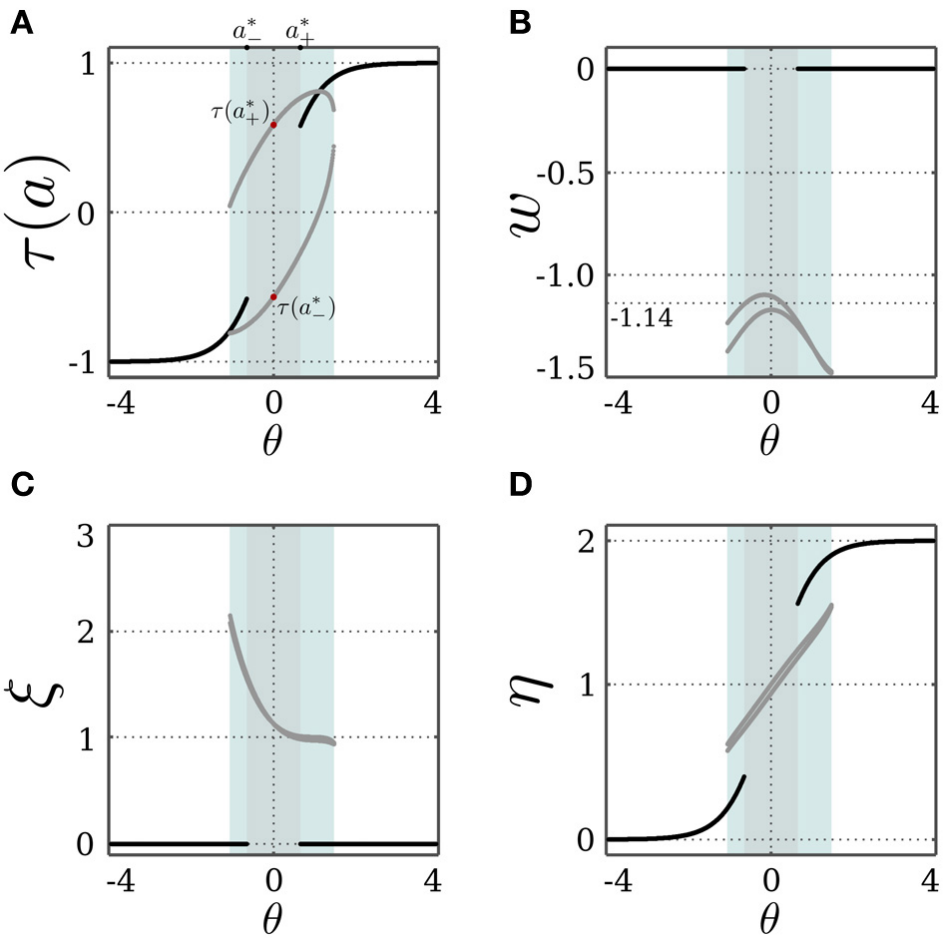
**Stable dynamics of a SR-neuron with inhibitory self-connection for varying bias**. Bifurcation diagrams of **(A)** the output τ(*a*), **(B)** the *negative* self-weight *w* = −ξ η, **(C)** the receptor strength ξ, and **(D)** the transmitter strength η for varying biasthe θ. The cyan-shaded area marks the domain at which the SR-neuron may *oscillate* with a period-2 between the two gray branches. The gray-shaded area marks the homeostatic bias domain θ ∈ (a^*^_−_, a^*^_+_) where the SR-neuron is globally oscillating. Outside of this domain, and depending on the initial conditions, the neuron may converge to the trivial fixed point (θ, 0, η_0_), corresponding to the black branches. **(A)** The red dots mark the oscillation in activity between a^*^_+_ and a^*^_−_ when θ = 0.

Figure 5 demonstrates that the SR-neuron *does* oscillate with period-2 on the bias domain (−0.95, 1.5) when *I* = 0. For zero bias, the self-weight oscillates due to the SR-dynamics with an average of *w* ≈ −1.14 < *w_c_* = −1, as is suggested by Equation (17). Interestingly, the oscillatory dynamics for non-zero bias allow the SR-neuron's output to reach average values that are different from the canonical τ(a^*^_±_) and the trivial τ(θ).

**Figure 6:**
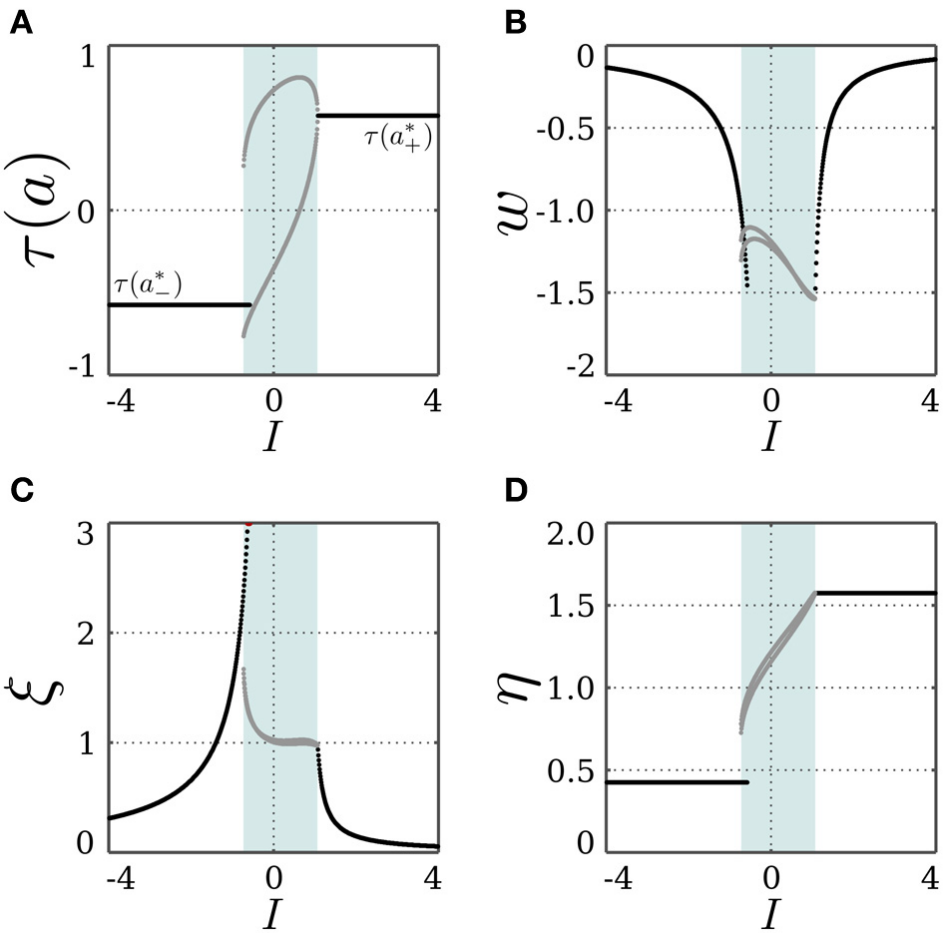
**Stable dynamics of a SR-neuron with inhibitory self-connection for a homeostatic bias and varying input**. Bifurcation diagrams of **(A)** the output τ(*a*), **(B)** the *negative* self-weight *w* = −ξ η, **(C)** the receptor strength ξ, and **(D)** the transmitter strength η for varying input *I* and a *positive* homeostatic bias θ = +0.5. The cyan-shaded area marks the domain at which the SR-neuron *oscillates* with a period-2 between the two gray branches. The neuron shows a narrow range of input at which the oscillatory and homeostatic activity are overlapping.

For a stationary input *I*, and a bias θ ∈ (a^*^_−_, a^*^_+_), a solution of Equation (16) may exist, and the SR-neuron acts as a homeostatic unit for a certain input domain. Also, since the bias is within the oscillation domain for no input, the SR-neuron should oscillate with period-2 for some input range around 0. In fact, as shown in Figure 6, the homeostatic domain overlaps with the oscillatory domain for a narrow input range.

### 3.2. Synaptic dynamics in the sensorimotor loop

In this section, we demonstrate for three examples how SR-neurons are able to operate successfully within the sensorimotor loop. On specific network structures, SR-neurons generate a desired behavior for coupled pendula, a hexapod walking machine, and a wheel-driven robot.

#### 3.2.1. Coupled reflex loops

Self-excitatory SR-neurons are good candidates for building oscillatory reflex loops. This was already shown in Pasemann (2013), where a single SR-neuron with excitatory self-connection was used to drive a pendulum with damping to oscillate with a constant amplitude. An angular position sensor is coupled to the reflex loop which drives the angle-controlled servomotor of the pendulum. Reflex loops generate smooth oscillatory movements which can be used for the control of limbs (von Twickel and Pasemann, 2007). There are two important mechanisms involved in the generation of these oscillations. First, the integration of properties of the body—the body's inertia in the case of pendula or limbs—and the environment by means of the sensorimotor loop. Second, the nonlinearity of the neural elements, leading to a hysteresis effect. Stated differently, oscillations do appear if the system can “jump” from one fixed point to another by following the slow transients generated by the inertia of the body. If there is no hysteresis but the sigmoid is steep as in Figures 1A,C, oscillations may appear, but with much smaller amplitudes, since there is no bistability interval to make the transients longer, and these oscillations will not be sufficient to provide the full swing of a limb for successful locomotion. In the case of an SR-neuron, hysteresis is provided by an excitatory self-connection (see Figure 4), which leads to bistable motor outputs. The time delay in the sensorimotor loop due to the physical characteristics of the body, its inertia namely, then causes the slow oscillations, referred to as *reflex oscillations*.

**Figure 7:**
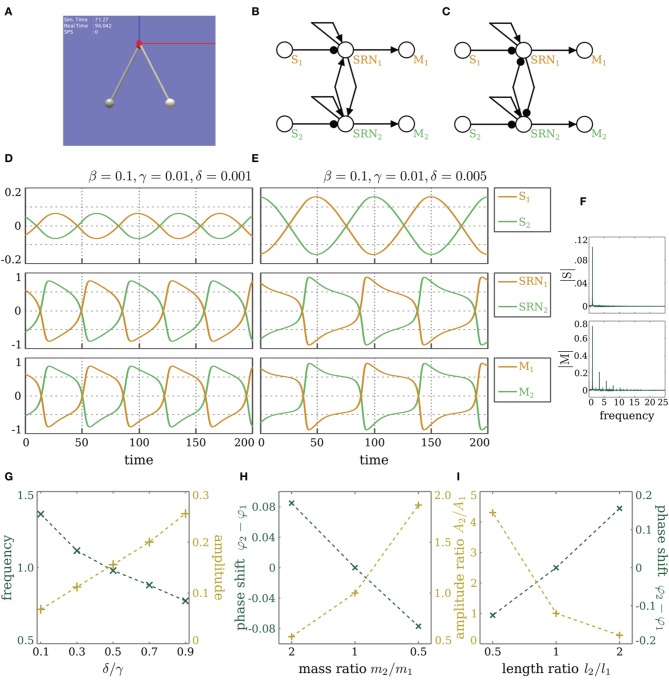
**Coupled reflex loops. (A)** Simulator of two identical pendula. The bob mass *m*_1,2_ = 0.2 kg and the rod length *l*_1,2_ = 0.5 m. **(B,C)** Coupled reflex loops for controlling the two identical pendula. Angular velocity sensors are linear buffers. The self-excitatory and the motor neurons are SR-neurons. **(B)** Lateral excitation leads to synchronization. **(C)** Lateral inhibitory leads to anti-synchronization. **(D,E)** Outputs of the angular velocity sensors, the SR-neurons, and the motor neurons of the two identical pendula, oscillating *anti-synchronously* due to *inhibitory* coupling. The parameters of the SRN-dynamics are set such that **(D)** δ/γ = 0.1, or **(E)** δ/γ = 0.5. **(F)** Fourier analysis of the signal coming from the sensor (top) and the motor (bottom) for δ = 0.001. **(G)** The effect of the quotient δ/γ on the amplitude and frequency of the oscillations. **(H,I)** Nonidentical pendula. The effect of changing **(H)** the bob mass ratio and **(I)** the rod length ratio on the phase shift between the two pendula and on the relative amplitude of their oscillation.

Before utilizing the SRN-dynamics and reflex loops for the locomotion of a hexapod walking machine, we demonstrate that the coupling of two such reflex loops leads to synchronization or anti-synchronization, depending on whether the coupling is excitatory or inhibitory. Coupling the hysteresis elements of two reflex loops by symmetric excitatory connections (or a unilateral connection for that matter) will enforce the synchronization of the resulting oscillations. Correspondingly, inhibitory coupling will result in anti-synchronization. Two pendula are driven by servomotors placed at each pendulum's pivot and are angle-controlled (see Figure 7A). Each servomotor is driven by a motor neuron whose output range (−1, +1) is mapped to the desired angle range (−180°, +180°). The desired angle is achieved through the servomechanism of position feedback, which applies a force of up to 0.5 N, until the error between the actual and desired angle is minimized. The parameters for the pendula are fixed to 0.2 kg for the bob mass and 0.5 m for the rod length. The angular position sensors are linear buffers, while the self-excitatory and the motor neurons are SR-neurons, as shown in Figures 7B,C.

Figures 7D,E demonstrate the dependence of the oscillation amplitude, and consequently its frequency, on the SR-neurons parameters. β and γ are fixed to 0.1 and 0.01, respectively. δ is either 0.001 (Figure 7D) or 0.005 (Figure 7E). The behavior of each pendulum is captured by its respective angular position sensor. By comparing the sensory signals, coming from the angular position sensors (top panel in Figures 7D,E), to that of the output of the motor neurons (bottom panel in Figures 7D,E), one notices that, despite the presence of damping, the pendula are oscillating harmoniously (sinusoidal motion with constant amplitude), although the outputs of the motor neurons show a different behavior. This can be confirmed by performing a Fourier analysis on the signals, which shows a single dominant frequency in the signal produced by the sensor, indicating that the pendulum generates a sinusoidal motion, i.e., a simple harmonic oscillation, while the motor produces multiple harmonies. This is illustrated in Figure 7F for δ = 0.001. One also observes that the amplitude of oscillation depends on the quotient δ/γ. For growing quotient δ/γ ≤ 1, the amplitude increases, and correspondingly, the frequency decreases (Figure 7G). For δ/γ > 1, the hysteresis domain widens to the point where the changing input is not enough for the dynamics to cross the bistable region, so it converges to one of the stable fixed points, and oscillations stop. These results, illustrated on the anti-synchronous case with lateral inhibition, also apply to the synchronous case with lateral excitation. The two cases are demonstrated in Movie S1.

Interestingly, for pendula with non-identical bob masses and rod lengths, one observes the emergence of phase-locking phenomena, but with differing oscillation amplitudes of the two pendula, as shown in Figures 7H,I and also in Movie S1. A mathematical analysis of this result is currently under development.

#### 3.2.2. Controlling a hexapod walking machine

**Figure 8:**
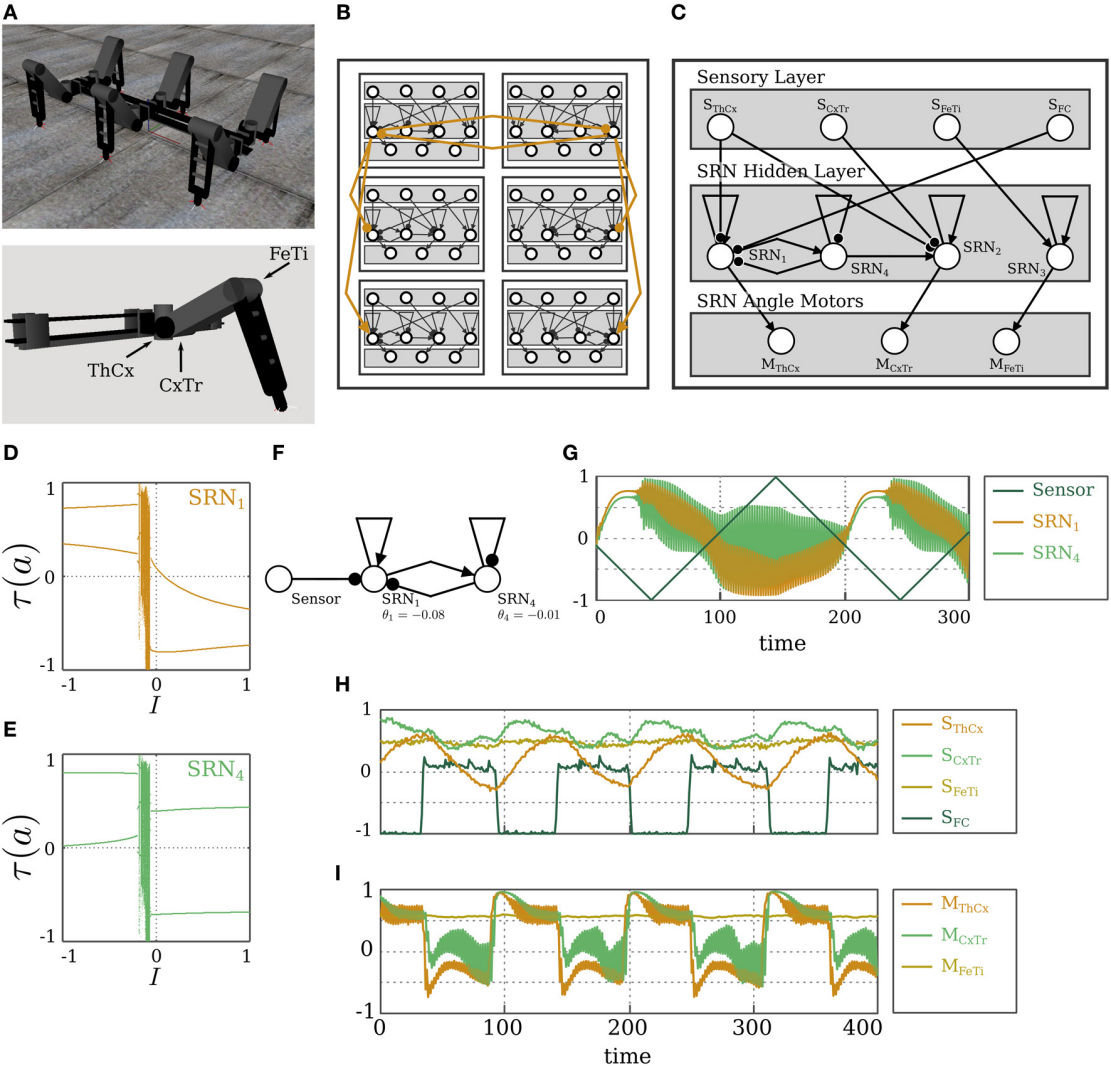
**Hexapod walking. (A)** The physical simulation of the hexapod walking machine Octavio (top), and of a single leg with the three joints marked (bottom). **(B)** The SRN-controller for the hexapod machine, highlighting the coupling scheme between the legs. **(C)** The SRN-controller of a single leg. **(D,E)** Bifurcation diagrams for varying sensory input *I* coming to the neuron *SRN*_1_. **(D)** The output of *SRN*_1_, and **(E)** the output of *SRN*_4_ in the leg module L1. **(F)** The oscillatory odd 2-ring network in the leg module L1. **(G)** The outputs of *SRN*_1_ and *SRN*_4_ for a sweeping sensory signal with a frequency comparable to that of the ThCx-joint oscillations. **(H)** Sensory and **(I)** motor signals of the left frontal leg module L1 during the hexapod walking.

It was demonstrated in Pasemann (2013) that reflex loops of SR-neurons can drive the three joints of a single leg to induce locomotion of the modular hexapod walking machine Octavio (von Twickel et al., 2012), shown in Figure 8A. Having observed that excitatory (inhibitory) coupling of SR-neurons in reflex loops leads to their synchronization (anti-synchronization), it follows that this method may be used to couple the neurocontrollers of single legs to get a walking behavior from the 18 degrees of freedom of the hexapod walking machine. For setting up a promising coupling structure, we assume that the protractor/retractor joint, named the ThCx-joint, of the left and right frontal legs, L1 and R1, respectively, gives the leading signals for the middle and hind legs (L2,R2 and L3,R3), and that the movement of these joints (of L1 and R1) needs to anti-synchronize. Thus, reflex loops of ThCx-joints of L1 and R1 are laterally coupled by inhibitory connections. The reflex loop of the ThCx-joint of the middle leg L2 (R2) receives an inhibitory synapse from the reflex loop of the ThCx-joint of L1 (R1), while the corresponding reflex loop of L3 (R3) receives an excitatory synapse from its counterpart in L1 (R1). This coupling scheme, shown in Figure 8B, should then lead to a typical *tripod gate*. The rationale behind this is as follows. The controller of each leg consists of three reflex loops. This entails that a leg could be considered as a high-dimensional reflex oscillator. According to the results from the previous section, coupling two reflex oscillators with an inhibitory connection would lead to their anti-synchronization, and with excitatory connection would lead to their synchronization. In other words, L1 and L3 would synchronize, due to the excitatory coupling between the two. L1 would also synchronize with R2, since the former is coupled to the latter by a chain of two inhibitory connections, which is equivalent to an excitatory coupling. The synaptic delay between L1, R2, and L3 is maximally two time steps, which has no effect and can be ignored, given the period of the reflex oscillations. This entails that the triplet (L1,R2,L3) would go through the *stance phase* simultaneously, while the anti-synchronous triplet (R1,L2,R3) would be in the *swing phase*, which results in a tripod gate.

Starting with a single leg reflex loop controller, and demanding the same controller structure for all the six legs, the described coupling scheme did not immediately lead to successful walking. To circumvent this, the evolution environment of the NERD Toolkit (Rempis et al., 2010) was utilized for evolving the structure further, and optimizing the bias values. The fitness function was given as “the distance walked in forward direction in a given number of time steps.” Regarding bias terms, a symmetry constraint was set to have identical left and right leg modules. As for the network structure, a constraint is set such that all legs are identical, and they follow the structure of L1. Other constraints, such as distance of the central body from ground or allowed joint angles, were not used in this case (also compare von Twickel et al., 2011, 2012). Text S1 outlines the details of the evolution process. Following evolution, the bias values of frontal, middle, and hind legs turned out to be different for achieving better forward walking. This is due to the fact that their task is different: frontal legs pull the body, while hind legs push the body. Figure 8B displays the complete modular neurocontroller. The resulting modules have identical structures for all legs due to the imposed constraints, and one of these modules is detailed in Figure 8C. SRN-parameters for this controller are set to β = 0.1, γ = 0.1, and δ = 0.2, which matches their values in the reflex loop controller of a single leg (Pasemann, 2013).

In addition to the simple reflex loops of the three joints from which evolution started, we find here an additional neuron *SRN*_4_ with inhibitory self-connection, which forms an odd 2-ring with the neuron *SRN*_1_ (the reflex loop of the ThCx-joint). This self-inhibitory neuron *SRN*_4_ and its connections were added by structure evolution. This additional structure induces period-2 oscillations, which at the first sight, might appear as superficial or destructive. However, all controllers that succeeded in achieving the forward motion of the body included this oscillatory neuron, and analysis shows that inhibiting these oscillations will result in a break down of walking. Figure 8F depicts the oscillatory odd 2-ring network, and Figures 8D,E show the bifurcation diagrams of its SR-neurons' output for a changing input signal. One observes that the module behaves as a period-2 oscillator over most of the input domain. It oscillates around positive amplitudes for negative inputs and around negative amplitudes for positive inputs. The asymptotic dynamics also shows a narrow regime of quasi-periodicity in the middle, which has no effect on behavior, since the dynamics passes over this domain for a short transitory period. This becomes clearer from Figure 8G, which illustrates the effect of a sensory signal sweeping over the interval [−1, 1] on the oscillatory module. The sensory signal sweeps over the interval with a frequency comparable to that of the ThCx-joint oscillations. This further highlights the dependence of the oscillation amplitudes on the sensory signal. We postulate that these oscillations are necessary for behavior, because they increase the range of admissible outputs. By having a changing mean value, which depends on the input strength, *SRN*_4_ allows for motor signals that are not restricted to the τ(a^*^_±_) values provided by reflex loops. Furthermore, the oscillatory effect of *SRN*_4_ is not seen anymore on the sensory signals coming from the joint angle sensors, as illustrated in Figure 8H. The oscillatory signal also has no direct effect on behavior in the sensorimotor loop. As demonstrated in Figure 8I, it only results in small amplitudes at the motors, and the effective motor signal corresponds to the mean value of these oscillations.

**Figure 9:**
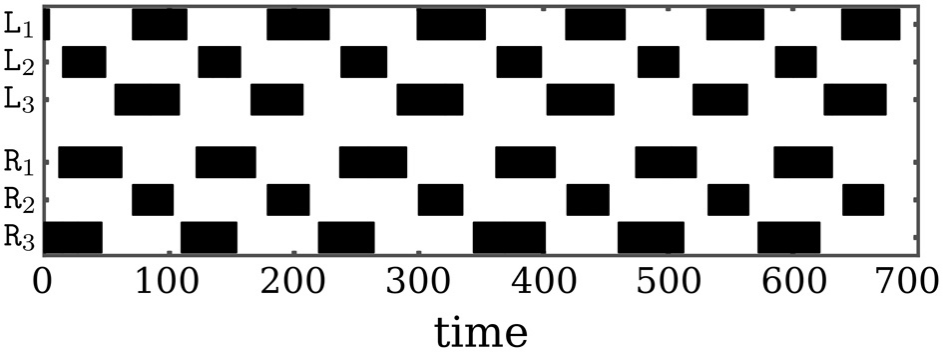
**Tripod gate of Octavio**. The walking pattern resulting from the neural control network of Octavio. Black regions mark the stance phase of the corresponding leg, which is the time span the foot is in contact with the ground.

Walking starts with the feet having ground contact. That the walking pattern is not a perfect tripod gate, but still represents a reasonably good walking behavior, can be read from Figure 9. As the walking pattern of Octavio in Figure 9 indicates, the stance phase of the middle legs are considerably shorter than those of the frontal and hind legs. Nevertheless, there is a uniform timing of the phases, so that walking on a flat surface is stable. That is, one notices that the stance phases of the triples (L1,R2,L3) and (R1,L2,R3) proceed almost periodically from one step to the next, which is a signature of stable tripod forward locomotion (see Movie S2 for demonstration).

In summary, although the suggested basic neural structure, i.e., the simple reflex loops, does not produce the desired behavior, an additional structure, even when adding oscillations, will generate this behavior. Here, it appears that walking is driven by mean values of fast oscillations. The amplitudes are small at the motors and integrated away by the body, as can be seen from the (noisy) sensory signals. One can also observe that inputs crossing bifurcation points, as is shown for instance in Figure 8G, do not derogate the desired behavior.

#### 3.2.3. Obstacle-avoidance with a wheel-driven robot

**Figure 10:**
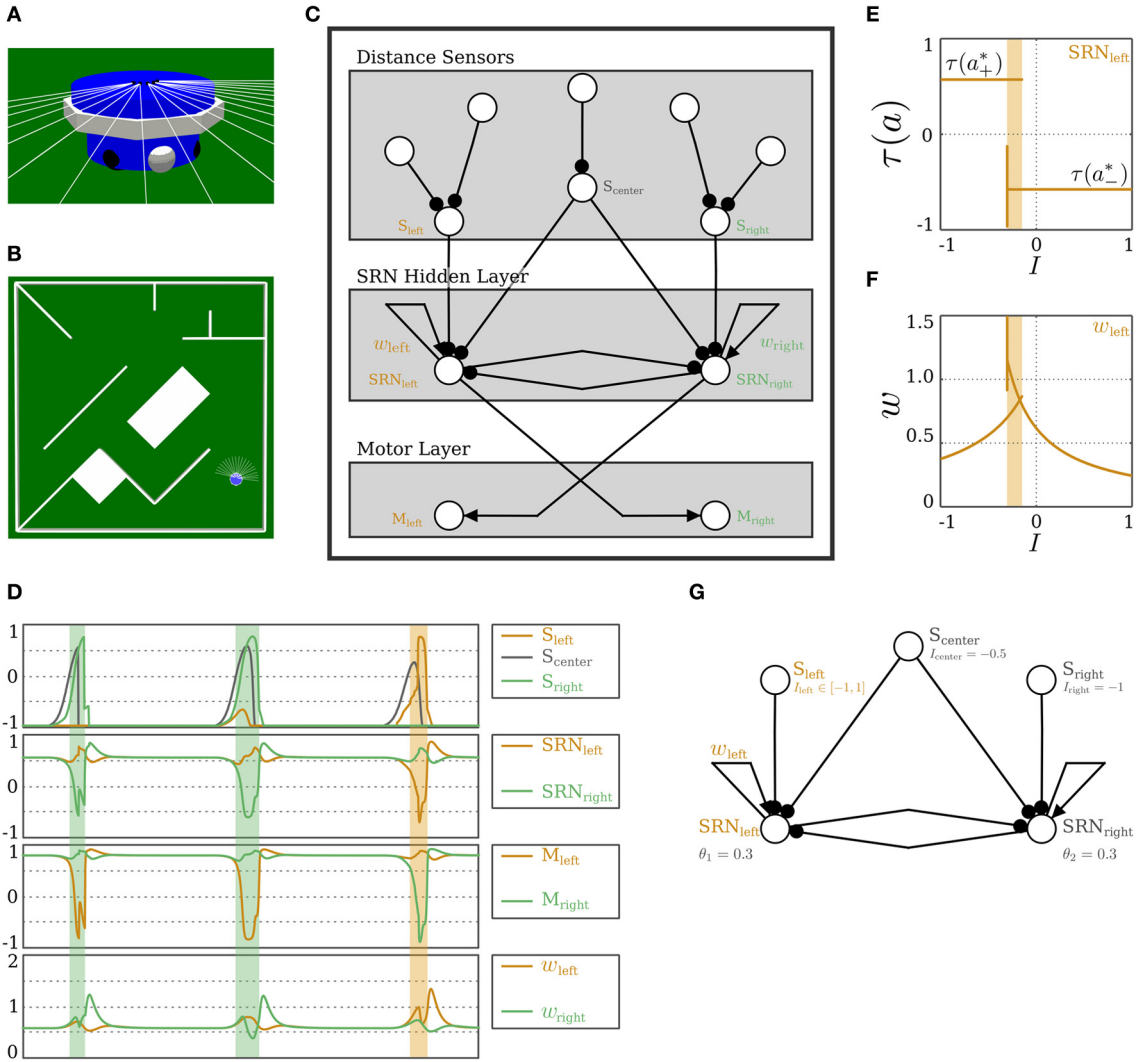
**Obstacle-avoidance with a wheel-driven robot. (A)** The two-wheeled robot Alice with distance sensors and wheels shown. **(B)** A typical navigation environment with obstacles. **(C)** A SRN-controller for obstacle-avoidance. Both sensory and motor layers have standard neurons. **(D)** Plots illustrating the dynamics of obstacle-avoidance behavior. From top to bottom: Output of the distance sensors; Output of the self-regulating neurons; Output of the motor neurons; Strength of the self-coupling of the SR-neurons. The shaded areas mark the time when *S*_left_ or *S*_right_ are sufficiently being stimulated, and are color-coded to match the side from which the obstacle is being approached. **(E,F)** Bifurcation diagrams for varying input from the sensor *S*_left_ of **(E)** the output τ(*a*) and **(F)** the self-weight *w* = +ξ η of *SRN*_left_. The shaded area marks the bistable domain. **(G)** The hidden layer of the obstacle-avoidance control network at which the bifurcations **(E,F)** are observed. A narrow corner approached from the left is emulated by stimulating the sensor S_center_ and varying the input from *S*_left_.

The SRN-dynamics is not restricted to the control of coupled reflex loops. We now show how a network of SR-neurons can be used by a wheel-driven robot (Figure 10A) to navigate its environment and avoid obstacles (Figure 10B). The wheel-driven robot is called Alice (see Figure 10A). Alice is endowed with five long-range distance sensors in the frontal part of the body, used for detecting obstacles. Each of Alice's two wheels is controlled independently by a motor neuron that drives a velocity-controlled servomotor. Each motor neuron's output range (−1, +1) is mapped to the corresponding servomotor's desired velocity range (−20°, +20°) per time step. The desired velocity is achieved through the servomechanism of position feedback, which applies a torque of up to 2 N·m, until the error between the actual and desired velocity is minimized. A preliminary example for a Khepera robot was also presented in Zahedi and Pasemann (2007), where the neurons had a different SRN-dynamics and a logistic sigmoidal nonlinearity, and a simpler neurocontroller was used. In what follows, we elaborate on the role of the current SRN-dynamics in achieving a successful obstacle-avoidance behavior, and we compare the behavior to the previous approach.

Figure 10C shows the control network using SR-neurons for obstacle-avoidance. It consists of three layers. The sensory layer assembles the five distance sensors into three groups corresponding to left, center, and right distance sensors, i.e., *S*_left_, *S*_center_, and *S*_right_, respectively. The input layer projects into a layer of hidden neurons of the self-regulating type. The hidden layer in its turn projects to the motor layer. The three sensor neurons and the motor neurons *M*_left_ and *M*_right_ are standard additive neurons with a hyperbolic tangent transfer function.

In order to understand the functioning of this network in controlling obstacle-avoidance, and the role of the self-regulating dynamics in achieving this, we look in more detail into the hidden layer. It consists of two SR-neurons: *SRN*_left_ and *SRN*_right_. Both are receiving input from *S*_center_. *SRN*_left_ is connected to the left-side distance sensors and in turn projects to the right motor. The reverse is true for *SRN*_right_. The SR-neurons are self-coupled with excitatory synapses. As shown in Figure 10D, an obstacle approached from the left side inhibits *SRN*_left_ and the sign of its output changes into negative. This in turn leads the velocity of the right wheel to become negative, which corresponds to a backward rotation of the wheel. Due to the lateral inhibition of *SRN*_right_ by *SRN*_left_, the left motor neuron *M*_left_ is excited, and the left wheel rotates faster in the forward direction. The combination of the backward rotation of the right wheel and the forward rotation of the left leads Alice to turn to the right and away from the left-side obstacle.

**Figure 11:**
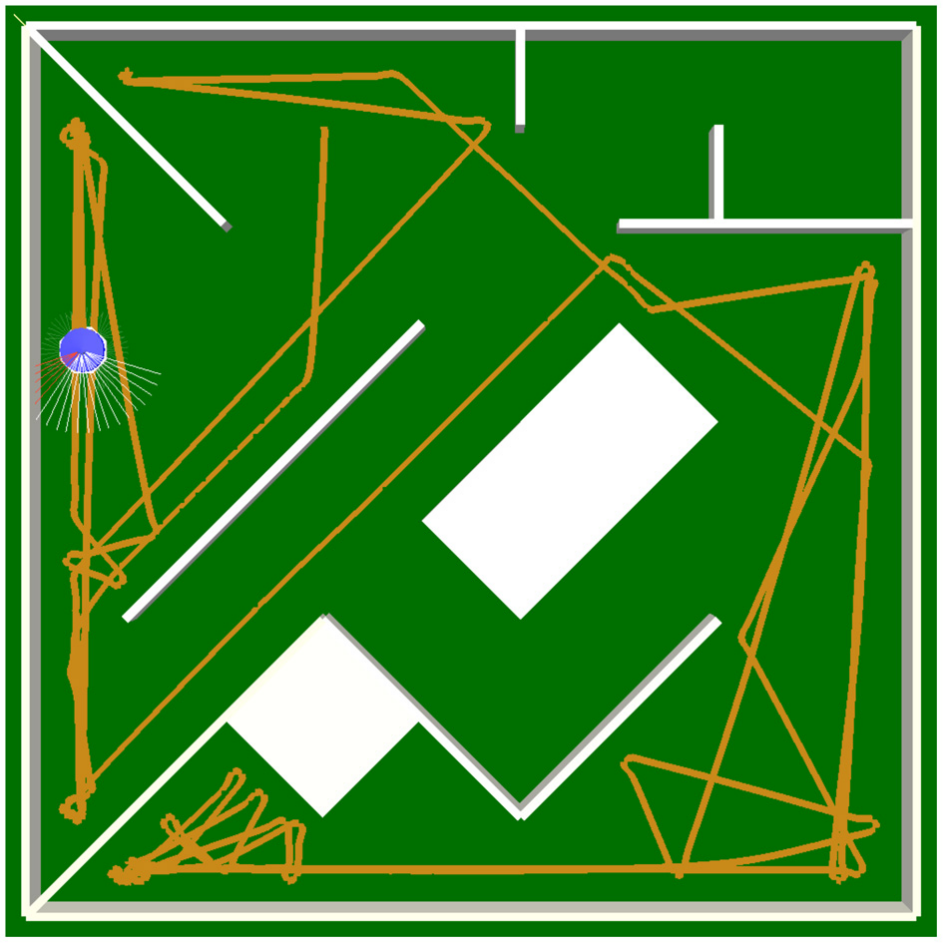
**Robot trajectory during obstacle-avoidance behavior**. The robot is capable of avoiding sharp corners, while being at a close proximity to the walls, due to hysteresis effects of the neurocontroller and the hyperbolic tangent nonlinearity. The latter allows the robot to stop and turn in place.

The switch of the sign of a self-regulating neuron in the hidden layer is particularly important when approaching a narrow corner. It is simply not sufficient for the output of the neuron to decrease due to the inhibition from the distance sensors. If this switch did not occur, Alice would turn right, but it would keep going forwards with less velocity, and it would not be able to avoid the sharp corner. In addition, the hysteresis effect resulting from the self-excitation allows the SR-neuron to memorize the history of its input, which is necessary for the avoidance behavior to continue in the same direction, preventing the robot from getting stuck (see Movie S3). Figures 10E,F show how the dynamics of *SRN*_left_ changes when a narrow corner is approached from the left (Figure 10G). The bifurcation diagram shows a hysteresis phenomenon where the neuron's output is bistable for a narrow range of input (recall the analysis of self-excitation above). The sign of the output *SRN*_left_ only changes when the input is strong enough to cross the hysteresis domain. Bistability, and the resulting hysteresis, which are necessary for behavior, cannot be explained by a particular component of the 6-dimensional dynamical system that is the hidden layer. The same network structure with no self-regulating connectivity could achieve the same effect if the weights were fine-tuned by hand or through evolution. For instance, it was shown in Hülse and Pasemann (2002) that with a similar controller, but with standard hyperbolic tangent neurons, the self-connections should be set above the critical value of *w_c_* = 1 for the hysteresis phenomenon to occur. Figures 10E,F show, however, that with SR-neurons, the phenomenon occurs without the self-connection crossing the critical value. These observations are also confirmed in the plots in Figure 10D. In other words, these properties *emerge* from the SRN-dynamics. In the previous study by Zahedi and Pasemann (2007), the different SRN-dynamics and neurocontroller were also capable of memorizing the history of the stimulus, allowing the Khepera robot to avoid narrow corners. However, due to the logistic sigmoidal nonlinearity being strictly positive, the robot was only capable of slowing down when turning away from narrow corners. On the other hand, the ability of the current model to generate a negative motor output allows Alice to turn in place, and as shown in Figure 11, to avoid more challenging obstacle scenarios, where the robot is at a close proximity to the walls and corners.

## 4. Discussion

We demonstrated that SR-neurons have a wide range of functions, depending on their bias terms and inputs coming from sensors or other neurons in the network. Without self-connection, they can serve as self-regulating units that are able to stabilize their activation around two desired outputs, which, in a way, correspond to low (a^*^_−_) and high (a^*^_+_) activity. For bias terms outside the interval (a^*^_−_, a^*^_+_), SR-neurons may get dysfunctional, i.e., their receptor strength converges to zero. Adding self-excitation to a neuron preserves the neuron's homeostatic properties, and introduces bistability, which allows the neuron to exhibit a hysteresis effect over a certain input range. A second operational mode of SR-neurons, due to self-inhibition, is that of a period-2 oscillator with varying and shifted amplitudes, depending on the bias and input.

Afterwards, we studied the properties of SR-neurons when operating in the sensorimotor loop. That is, SR-neurons are driven by changing sensory inputs, and they generate motor signals accordingly, which in their turn drive the actuators of an animat. From experiments with pendula, single legs (Pasemann, 2013), and hexapod walking machines, one concludes that SR-neurons are suitable for coupling reflex loops, because desired sensory inputs do change frequently or are oscillating. As a result, and due to SRN-dynamics, appropriate mean values of synaptic efficacy adjust themselves properly. However, examples from networks controlling wheel-driven robots demonstrate that the function of SR-neurons is not restricted to reflex loops. Even if sensory inputs are not often changing, as is the case when no obstacles are present, mean values of the synaptic efficacies self-adjust, depending on the connectivity and the bias values, so that a desired behavior is achieved. It is worth noting that in the example of the wheel-driven robot, motor neurons are not self-regulating. However, choosing them to be self-regulating leads qualitatively to the same behavior.

The SR-neuron with excitatory self-connection is of particular importance for the control of an animat in the sensorimotor loop. The hysteresis effect, which such a module exhibits, provides the neuron with a working memory of the stimulus history, which allows it to produce oscillatory output. The period of these reflex oscillations depends on the width of the hysteresis domain, which is a function of the SR-neuron's parameters. This was the basis for generating the locomotion behavior of the hexapod walking machine. This dependence on input history also allowed the wheel-driven robot to turn in place and away from sharp corners by “remembering” the direction of the obstacle long enough to swing away from it. An SR-neuron with excitatory self-connection is a particular instance of a class of systems that exhibit bistability, and as a corollary, hysteresis. Namely, every ring of standard sigmoidal neurons undergoes a bifurcation for some values of the weights and biases, if and only if the number of inhibitory synapses is even, which leads to the existence of two fixed point attractors (bistability), in addition to coexisting periodic attractors (Pasemann, 1995). The bistability phenomenon is also relevant for genetic networks, and is shown to exist in these systems under similar conditions (Angeli et al., 2004). The significance of SRN-dyanamics is that it pushes the neuron's parameters *autonomously* toward the bistable regime, allowing it to implement a form of short-term plasticity, and the resulting working memory of input history (Zucker and Regehr, 2002; Abbott and Regehr, 2004; Mongillo et al., 2008).

The design of the SR-neurons with two operating points provides a natural implementation of the principles of *step mechanisms* and *ultrastability*, suggested by Ashby (1960) as main ingredients of adaptive behavior. These concepts are better explained through the example of obstacle-avoidance by the wheel-driven robot. The essential variables of this system are the readings of the distance sensors, which should remain close to their minimum for the survival of the robot. When the stability of the moving-forward behavior is broken, due to the approach of an obstacle from the left, it triggers a change in the value of a step mechanism implemented in the left SR-neuron by the SRN-dynamics, while no change occurs at the right SR-neuron, i.e., a new behavior, turning-right, becomes stable. In other words, while the actions of the robot are continuous, only four stable modes of behavior are identified by the two step mechanisms provided by the two SR-neurons. These allow the robot to keep its essential variables within the desired range: the robot's behavior is ultrastable.

Synaptic plasticity with homeostatic regulation has been applied several times in the context of *evolutionary robotics* (Di Paolo, 2000; Harvey et al., 2005; Santos et al., 2010), and has been related to Ashby's theory (Ashby, 1960) as well. In these studies, neurocontrollers for autonomous robots are evolved such that each synapse is assigned a synaptic plasticity rule from a set of possible variants of Hebbian plasticity. Synaptic dynamics get activated only when neural output diverges from a selected homeostatic domain. Others investigated comparable mechanisms where homeostasis was also discussed in the context of walking behavior (Hoinville and Hénaff, 2004; Hoinville et al., 2011). Our approach differs from those in that homeostatic stability is achieved using a single plasticity mechanism, and in that it is written completely in dynamic terms. The SRN-dynamics is also related in part to the BCM theory (Bienenstock et al., 1982; Cooper et al., 2004). Both the BCM rule and SRN-dynamics achieve stability of synaptic weights through a quadratic dependence on postsynaptic activity, and on a threshold that separates the regimes of synaptic depression and potentiation. However, unlike BCM learning, it is not necessary for the threshold a^*^_±_ of SRN-dynamics to be sliding. This is due to the fact that homeostatic stability, as is the case in Triesch (2007), is explicitly implemented in the receptor dynamics. However, the SRN-dynamics differs functionally from the BCM rule, in that the latter is a learning rule, while the former is not.

Obstacle-avoidance with wheel-driven robots is a benchmark task in neural control, and successful controllers were found either through synaptic plasticity of the weights that connect sensors to built-in reflexes (Harter and Kozma, 2005), the homeostatic regulation of a GasNet control networks with artificial chemicals during evolution (Vargas et al., 2009), or maintaining homeostasis by modulating the random reconfiguration of the conroller's parameters by artificial hormones (Pitonakova, 2013). The SRN-dynamics control of the wheel-driven robot does not incorporate learning as in Harter and Kozma (2005); and unlike (Vargas et al., 2009; Pitonakova, 2013), where the robot has to carry out multiple tasks concurrently, Alice's behavior is restricted to obstacle-avoidance. However, in these studies, the neurocontrollers are derived and tested in spacious maze-like environments (Harter and Kozma, 2005), or in a featureless rectangular arena (Vargas et al., 2009; Pitonakova, 2013), and would not avoid narrow impasses or sharp corners. On the other hand, neural control with SR-neurons exploits the full potential of the recurrent neural network, as well as the bistability resulting from the synaptic dynamics, thus succeeding where other controllers would fail.

A hallmark of the current study is the derivation of a stable forward walking behavior of a hexapod with 18 degrees of freedom, corresponding to the 18 joints of the insect-like robot Octavio. Beer and Gallagher (1992) used an evolutionary process to derive a neurocontroller to achieve stable walking of a hexapod. While that hexapod also contained 18 degrees of freedom, it only had 6 joints. Achieving stable behavior of a quadruped or a hexapod with multiple joints per leg is far from trivial. For instance, Shim and Husbands (2012) used intrinsic chaos of weakly-coupled central pattern generators to search for a neurocontroller of a quadruped with eight degrees of freedom, and later stored the successful controllers in the connections between the oscillators, using a form of synaptic plasticity. While the same strategy led to a stable forward locomotion of a swimming robot, Shim and Husbands (2012) reported that the behavior of the quadruped broke after some time. However, a stable 18-joints hexapod forward locomotion is achieved using Walknet (Schilling et al., 2013a). Walknet allows for a variety of behaviors and extensions to match the behavioral repertoire of a stick insect (Schilling et al., 2013b). This flexibility comes with the price of a highly complex and heavily engineered controller with many non-neuronal elements. On the other hand, the SRN-controller of Octavio provides from simple design intuitions, and a small contribution from evolution (a single hidden neuron for each leg), a minimal architecture with dynamic synapses that is, to this point, unprecedented.

From the experiments described here, it is obvious that an effective control also depends on convenient SR-parameters, which were currently picked by hand. However, these parameters can, in principle, be optimized using evolutionary techniques provided, for instance, by the NERD Toolkit (Rempis et al., 2010). The same applies to bias terms. An alternative is to find suitable bias dynamics, which is a topic of current research. Often, there are reasonable constraints on the structure of more complex neural controllers. The NERD evolution environment allows the use of functional substructures, symmetry constraints, modularization, specific synaptic communication lines or nerve bundles, and a variety of different neuron types, such as sensor neurons, bias neurons, standard neurons, and SR-neurons (Rempis et al., 2010). These capabilities were used, for instance, for the control of forward/backward locomotion of a single leg (Pasemann, 2013), and the current control of locomotion of the hexapod walking machine.

In addition, the connectivity of the network is equally essential for the synaptic dynamics for deriving an effective control. Instead of finding solutions in a high-dimensional real-valued parameter space, evolution can be utilized to find *only* those (−1, 0, +1) connectivity structures on which the SRN-dynamics leads to a satisfactory behavior. However, finding the real-valued bias terms remains a bottleneck, due to the lack of an appropriate bias dynamics. An alternative approach to evolution in refining an agent's behavior is the introduction of proprioceptive units that dissipate artificial neuromodulatory signals. These units are placed within preconfigured networks that are separate from the robot's neurocontroller, and are responsible for monitoring the robot's behavior. For instance, a monitoring network may be responsive to the robot's failing to avoid an obstacle, or approach food sources. When either undesired behavior occurs, the monitoring network stimulates its corresponding proprioceptive unit. The latter would then release a signal that initiates the learning of SR-parameters, bias terms, or connectivity structure *during* the lifespan of the robot. The neuromodulatory signals stop, when the robot's behavior is appropriate and the monitoring networks are deactivated (Rempis et al., 2013).

In the context of connectivity, an interesting property of a SR-neuron is that it can turn off its input by reducing its receptor strength down to zero, thereby becoming a “dead neuron.” This fact may be used to facilitate the evolution of effective connectivity structures. For example, starting with a fully connected network, the bias term of a neuron may enter the dead neuron domain, either through evolution or by accommodating *bias dynamics*. Taking such a SR-neuron, which can no longer contribute to a behavior-relevant synaptic dynamics, out of the network will correspond to a mechanism similar to a programmed death of a cell and it will prune the network structure.

## Author contributions

Conceived and designed the experiments: Hazem Toutounji and Frank Pasemann. Performed the experiments: Hazem Toutounji and Frank Pasemann. Analyzed the data: Hazem Toutounji and Frank Pasemann. Wrote the manuscript: Hazem Toutounji and Frank Pasemann.

## Funding

This research was partially funded by the German Research Foundation (DFG) priority program 1527.

### Conflict of interest statement

The authors declare that the research was conducted in the absence of any commercial or financial relationships that could be construed as a potential conflict of interest.

## Supplementary material

Experiments are performed using the NERD Toolkit (Rempis et al., 2010), an open-source software that can be used under an extension of the GNU General Public Licence (GPL). The Software is available at: http://www.ultopia.de/drupal/nerddoc/ The following are provided as supplementary material:

Text S1 **Evolving a neurocontroller for hexapod locomotion**.

Movie S1 **Corresponding to Figure 7, coupled reflex loops**.

Movie S2 **Corresponding to Figure 8, hexapod forward walking, tripod gate**.

Movie S3 **Corresponding to Figure 9, obstacle-avoidance with wheel-driven robot**.
