# Supplementary material for: Behavior control in the sensorimotor loop with short-term synaptic dynamics induced by self-regulating neurons
Source: Front Neurorobot. 2014 May 23;8:19. doi: 10.3389/fnbot.2014.00019 (PMC4033235; doi:10.3389/fnbot.2014.00019)
Supplement: Text S1 — Evolving a neurocontroller for hexapod locomotion. [file DataSheet1.PDF]

Frontiers in Neurorobotics

Research Article
13 May 2014

Supplementary Material: Behavior control in the sensorimotor loop with short-term synaptic dynamics induced by self-regulating neurons

Hazem Toutounji [1,*] and Frank Pasemann [1]

[1] *Institute of Cognitive Science, University of Osnabrück, Osnabrück , Germany*

Correspondence*:
Hazem Toutounji
Institute of Cognitive Science, University of Osnabrück, Albrechtstr. 28, Osnabrück, 49076, Germany, htoutounji@uni-osnabrueck.de

1 EVOLVING A NEUROCONTROLLER FOR HEXAPOD LOCOMOTION

The neurocontroller for forward locomotion of the Hexapod walking machine OCTAVIO was evolved using the *NERD Toolkit* (**Rempis et al.**, 2010), an open source software for research in evolutionary robotics and neurorobotics. The NERD Toolkit implements the *Interactively Constrained Neuro-Evolution* method (ICONE) (**Rempis**, 2012), which allows the experimenter to adjust parameters during the evolution process, and to reduce the search space by imposing constraints on the admissible network structures.

The evolution process proceeds as follows. First, an initial population of neurocontrollers is created. At each generation, individuals are evaluated according to a given fitness function. Then, an elite of the population is passed directly to the next generation. The population reaches its full capacity through a selection strategy among the individuals of the last generation. This is followed by adaptation operations on the resulting population that involves modifying the bias terms and introducing new neurons and synapses, depending on the imposed constraints. The experimenter may then decide to change certain parameters of the evolution process. **Supplementary Table 1** summarizes the relevant parameters and their values during the initial and final stages of the evolution process. The following sections elaborate on the different elements of the evolution process by which the successful neurocontroller of OCTAVIO was found.

1.1 INITIAL POPULATION AND EVOLUTION CONSTRAINTS

Each individual has the single leg reflex loop controller structure of its frontal left leg `L1` cloned to all six legs. The single leg controller consists of 3 positively self-coupled hidden SR-neurons that are fixed during evolution. Evolution then starts with 200 individuals. Regarding bias terms, a symmetry constraint was set to have identical left and right leg modules. As for the network structure, a constraint is set such that all legs are identical, and they follow the structure of `L1`. These constraints were enforced during the whole evolution process. In the initial generation, bias values of the neural modules of the legs `L1`, `L2`, and `L3` were randomized to ± 0.1, and then copied to those of `R1`, `R2`, and `R3`, in order to preserve the lateral symmetry constraint.

Supplementary Table 1. Parameters of the evolution process. Parameters changed by the experimenter at different points of the evolution process. The initial values of the parameters are shown along with the values at the end of evolution.

**Parameter**	Initial stage	Final stages
Population size	200	100
Initial bias values	± 0.1	
Evaluation time	1000	5000
Number of tries	1	5
Elite individuals	10	3
Tournament size	5	13, down to 5 later
Bias mutation probability	0.1	0.1
Bias range	$[-0.3, +0.3]$	$[-0.3, +0.3]$
Distribution of bias change	$\mathcal{N}(0, 0.01)$	$\mathcal{N}(0, 0.01)$
Maximum number of hidden neurons	5	
Probability of inserting or deleting a neuron	0.01	0
Maximum number of new synapses	10	
Probability of inserting or deleting a synapse	0.1	0

1.2 EVALUATION AND SELECTION

At each generation, the performance of all individuals is evaluated for several *tries* by the fitness function given by “the distance walked in forward direction in a given number of time steps”. The final fitness of an individual is the average fitness across tries. Introducing several tries aims at evaluating an individual for changing neural initial conditions and different noise in the sensors and motors. The evolution process is split into several stages regarding evaluation time. It starts at 1000 steps and a single try for an initial phase of several generations, after which, evaluation time is increased by the experimenter, and it reaches 5000 steps, 5 tries each, at the final stages of evolution. The decision to increase the evaluation time and the number of tries is mainly to ensure smoother and regular locomotion. It may also be due to the plateauing of the population overall fitness, following the eradication of individuals that fail to produce substantial forward locomotion in the shorter allotted evaluation time. In addition to increasing the evaluation time, the experimenter reduces the population size down to 100 individuals at the final stages of evolution.

After evaluation, specifying the next generation involves selecting a small *elite* of the fittest 3 individuals (the fittest 10 at the first stage). Then, the population is completed to its capacity using a *tournament selection strategy* (**Miller and Goldberg**, 1995), which goes as follows. A subpopulation of 5 individuals at the beginning and up to 13 individuals in intermediate stages is randomly selected with replacement. The fittest individual of this subpopulation wins the tournament and is passed to the next generation. This controls the distribution of fitness values of a population during evolution.

1.3 ADAPTATION

Before proceeding to the next generation, all individuals undergo three adaptation processes: inserting and deleting neurons, inserting and deleting synapses, and bias mutation. Probabilities for these operations are controlled by the experimenter.

The maximum number of allowed hidden neurons per leg module is 5 (maximally, 2 new neurons). Neurons are added to the hidden layer of the neural module of `L1`. Each of the potential 2 neurons has a 0.01 initial probability of creation. The maximum number of new synapses is 10, and each has 0.1 initial probability of creation. Both sensory and hidden neurons are potential sources of newly added synapses, while only hidden neurons are potential targets, all with uniform probability. The weight of the added synapse is then randomized to ± 0.1, and is now subject to the SRN-dynamics. After the first initialization,

a synapse never changes its sign. The deletion of neurons and synapses use the same insertion parameters. The original elements (the three hidden neurons and their efferents and afferents) are protected from deletion.

The bias terms of the hidden and motor neurons of the three left legs are all potential candidates for mutation by a Gaussian random walk. The bias of each neuron is selected for mutation with 0.1 probability. If a neuron is selected, its bias is adjusted by a random value drawn from a normal distribution with 0 mean and 0.01 variance. All bias terms are truncated to remain in the range $[-0.3, +0.3]$. After mutation, the new network structure is cloned to the other legs, and bias terms of the neurons from the left legs are copied to their contralateral counterparts to preserve the constraint.

After a successful structure with high fitness is found, the probabilities of inserting new synapses and neurons is set to 0 by the experimenter, so that evolution is focused on refining the bias terms.

REFERENCES

Miller, B. L. and Goldberg, D. E. (1995), Genetic algorithms, tournament selection, and the effects of noise, *Complex Systems*, 9, 3, 193–212

Rempis, C., Thomas, V., Bachmann, F., and Pasemann, F. (2010), NERD - Neurodynamics and Evolutionary Robotics Development Kit, in N. Ando, S. Balakirsky, T. Hemker, M. Reggiani, and O. Stryk, eds., Simulation, Modeling, and Programming for Autonomous Robots (Springer Berlin Heidelberg), Lecture Notes in Computer Science, 121–132, doi:10.1007/978-3-642-17319-6_14

Rempis, C. W. (2012), Evolving Complex Neuro-Controllers with Interactively Constrained Neuro-Evolution, Ph.D. thesis, Osnabrück University
